# When It's Heavier: Interfacial and Solvation Chemistry of Isotopes in Aqueous Electrolytes for Zn‐ion Batteries

**DOI:** 10.1002/anie.202300608

**Published:** 2023-03-10

**Authors:** Xuan Gao, Yuhang Dai, Chengyi Zhang, Yixuan Zhang, Wei Zong, Wei Zhang, Ruwei Chen, Jiexin Zhu, Xueying Hu, Mingyue Wang, Ruizhe Chen, Zijuan Du, Fei Guo, Haobo Dong, Yiyang Liu, Hongzhen He, Siyu Zhao, Fangjia Zhao, Jianwei Li, Ivan P. Parkin, Claire J. Carmalt, Guanjie He

**Affiliations:** ^1^ Christopher Ingold Laboratory Department of Chemistry University College London 20 Gordon Street London WC1H 0AJ UK; ^2^ Department of Chemical Engineering University College London London WC1E 7JE UK; ^3^ Institute of Technological Sciences Wuhan University Hubei Wuhan 430072 P. R. China; ^4^ Institute of Materials Science Technische Universität Darmstadt 64287 Darmstadt Germany

**Keywords:** Interface, Solvation structure, Isotopes, Zinc-ion Batteries

## Abstract

The electrochemical effect of isotope (EEI) of water is introduced in the Zn‐ion batteries (ZIBs) electrolyte to deal with the challenge of severe side reactions and massive gas production. Due to the low diffusion and strong coordination of ions in D_2_O, the possibility of side reactions is decreased, resulting in a broader electrochemically stable potential window, less pH change, and less zinc hydroxide sulfate (ZHS) generation during cycling. Moreover, we demonstrate that D_2_O eliminates the different ZHS phases generated by the change of bound water during cycling because of the consistently low local ion and molecule concentration, resulting in a stable interface between the electrode and electrolyte. The full cells with D_2_O‐based electrolyte demonstrated more stable cycling performance which displayed ∼100 % reversible efficiencies after 1,000 cycles with a wide voltage window of 0.8–2.0 V and 3,000 cycles with a normal voltage window of 0.8–1.9 V at a current density of 2 A g^−1^.

## Graphical abstract

The electrochemical effect of isotope of water is introduced into a Zn‐ion battery electrolyte to control severe side reactions and massive gas production. Due to the low diffusion and strong coordination of ions in D_2_O, the possibility of side reaction is decreased, resulting in a broader electrochemically stable potential window, less pH change, less zinc hydroxide sulfate (ZHS) generation and pure phase ZHS precipitation during cycling.

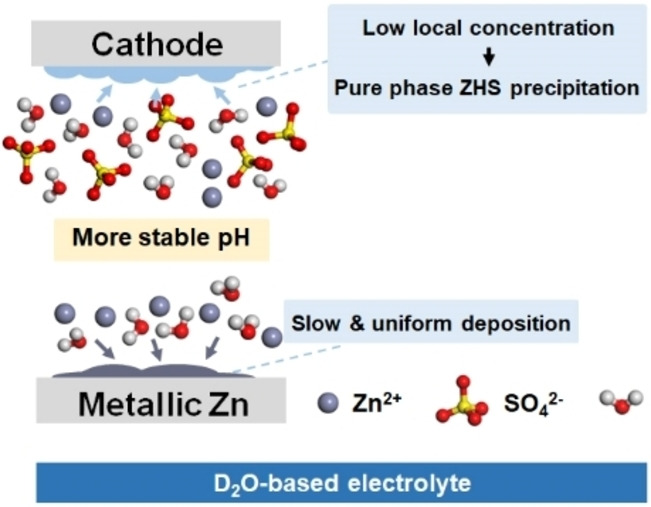

## Introduction

Aqueous electrolytes have been viewed as promising components for electrochemical energy storage application due to their low cost, ease of usage, and safety. With the advantages of excellent industrial adaptability and high energy density, aqueous Zn‐ion batteries (ZIBs) have been increasingly studied among the aqueous energy storage technologies in recent years.^[1]^ When the water is utilized as the electrolyte in ZIBs, it confers a high ionic conductivity because of large acceptor number (AN, 54.8) and donor number (DN, 18) as well as the high dielectric constant (78 at 25 °C).^[2]^ However, the practical application of aqueous electrolytes is restricted by their narrow electrochemically stable potential window of 1.23 V for pure water, due to the hydrogen evolution reaction (HER) and the oxygen evolution reaction (OER).^[3]^ To provide a wide operating voltage range for aqueous ZIBs, neutral salts, particularly sulphates with significant hydration with water molecules to create an energy barrier for water electrolysis, have been produced.^[4]^ On the basis of this salt‐dependent hydration mechanism, recent research in aqueous electrolytes has increased the electrochemical window (EW) by modifying the solvation structure, property, and concentration of the salt component of aqueous electrolytes.^[5]^ On the other hand, the complex side reactions in ZIBs seriously interfere with the stable cycle, which has become a great challenge for aqueous energy storage technology.^[6]^ Researchers have made lots of efforts developing various organic and inorganic electrolyte additives to alleviate the side reactions, so as to alleviate the gas production, pH changes and the generation of side productions, such as zinc hydroxide sulfate hydrate (ZHS) during the cycle.^[7]^ From the perspective of industrialization, the high‐cost production lines also require the introduction of additives that are easy to store and have limited influence on the battery charge and discharge behavior.

Apart from the electrolyte additive, the application of isotope effect in electrochemical energy storage technology is another promising direction to increase the potential window and cycling stability. Prof. Frederick Soddy used the term “isotope” in 1913 to describe two or more atoms that share the same atomic number (number of protons/electrons) but have distinct nucleon numbers (mass numbers) because they have different numbers of neutrons in their nucleus.^[8]^ These isotopes have the same chemical element but distinct atomic masses. The nuclear charge and electron configuration of an atom are determined by the number of protons, and the stable (non‐radioactive) isotopes of an element and their compounds typically exhibit comparable physicochemical characteristics.^[9]^ However, the isotope effect, which is caused by different mass and nuclear spin of the isotopes, can affect the thermodynamic and kinetic parameters of a chemical reaction, such as the equilibrium constant, and can result in differences in the physical properties of an element or compound (such as density, vapor pressure, and melting point).^[10]^ As there is a significantly greater mass difference between the isotopes for the light elements, the isotope effects are more pronounced. The two stable natural isotopes of hydrogen, protium (^1^H) and deuterium (^2^H or D), are the lightest elements in the Periodic Table.^[11]^ Protium and deuterium both have one proton, but deuterium contains an extra neutron in its nucleus, increasing its atomic mass by roughly 100 %. The hydrogen element and its derivatives exhibit strong isotope effects, which are explained by the enormous difference in atomic mass. Deuterium oxide (D_2_O, often known as heavy water) exhibits noticeably different physicochemical features when compared to protium oxide (H_2_O, or water), as seen in Table S1. From the perspective of D_2_O molecular, the isotopic effect is greatly influenced by intermolecular hydrogen bonding. The hydrogen bonds between H_2_O molecules are longer and less angular than those between D_2_O molecules due to the significant larger quality of D and the D‐bond energy of D_2_O is 1 kJ mol^−1^ larger than H‐bond energy of H_2_O based on the previous studies.^[3]^ With two lone pairs of electrons and two hydrogen atoms, a water molecule can interact with four surrounding water molecules to form a tetrahedral hydrogen‐bond network. Compared with H_2_O, the tetrahedron formed by D_2_O is more symmetrical, which is favorable for the formation of D‐bonds. Additionally, D_2_O shows a higher dissociation energy (E_d_) than H_2_O because the two molecules have the same total potential energy.[^3^, ^12^]

In this study, the electrochemical effect of isotope (EEI) is applied to the electrolyte of ZIBs for the first time to alleviate the side reaction and enhance the stability during the cycle. From a kinetic study perspective, molecular dynamics (MD) simulations of EEI in D_2_O and H_2_O were first performed and the low diffusion coefficient of ions in D_2_O was discovered to demonstrate the promising potential of D_2_O‐based electrolytes in ZIBs. Due to the low diffusion coefficient and strong coordination of ions in D_2_O, the probability of side reactions in the battery is reduced, exhibiting a wider EW, less pH variation, and less ZHS generation during cycling. More notably, the D_2_O environment can mediate the phase of ZHS during cycling. Here, we found for the first time that D_2_O suppresses the generation of different phases of ZHS induced by the change of bound water in ZHS during cycling, providing a more stable electrode‐electrolyte interface. The full cells with D_2_O‐based electrolytes exhibited more stable cycling performance than H_2_O‐based electrolytes, and exhibited ∼100 % reversible efficiencies after 1,000 cycles with a voltage window of 0.8–2.0 V and 3,000 cycles with a normal voltage window of 0.8–1.9 V.

## Results and Discussion

**Figure 1:**
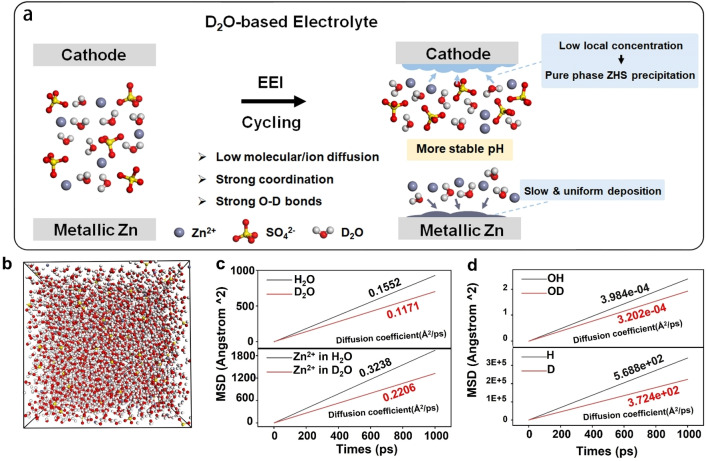
(a) Schematic diagram illustrating the influence from EEI of D_2_O. (b) The configuration of the electrolyte. (c) The mean square displacements (MSD) of Zn^2+^, H_2_O, and D_2_O in the corresponding electrolyte. (d) The MSD of the OH, OD, H and D on the surface of the metallic Zn.

As shown in Figure 1a, the D_2_O‐based electrolyte alleviates the side reactions of ZIBs during cycling and can be attributed to three aspects: low diffusion of molecules/ions, strong coordination between ions and D_2_O, and strong O−D bonds. From the perspective of the anode, the low diffusion of Zn^2+^ in D_2_O slows down the formation of Zn dendrites, making the interface formed by Zn^2+^ deposition flat. At the cathode, the EEI of D_2_O regulates the interface between the cathode and the electrolyte by controlling the bound water in the ZHS structure and the amount of ZHS. First‐principles calculations were performed to reveal the underlying mechanism when different solutes were adopted. As the growth of Zn dendrites and LDH is often closely related to the OER and HER on the anode surface, we first investigated the thermodynamic behavior on the metallic Zn anode surface. As evidenced by Figures S1a and b, it is clear that the adsorption of H, D, or OH, OD on the surface of Zn remains essentially the same. As the adsorption of H or OH on the electrode surface is the key step in the HER and OER, respectively, the adsorption energy and differential charge density difference diagrams provide sufficient evidence that there is no significant difference in the thermodynamic response of the two different electrolytes on the surface of the electrodes. We also performed the MD for the investigation of the bulk phase of the electrolyte in Figure S1c. However, no difference in the radial distribution function was indicated. The results fully demonstrate that despite the change in the electrolyte, there is no change in the thermodynamic behavior of the system.

Further, the behavior differences between D_2_O and H_2_O in ZIBs were investigated from a kinetic point of view. We make the long‐enough MD simulation to understand bulk electrolytes and their influence on the surface of the anode. As presented in Figure 1c, the diffusion coefficient of Zn^2+^ in the D_2_O and H_2_O are 0.22 and 0.32, respectively, which indicates the drop in the ionic conductivity. Furthermore, the diffusion coefficient of D_2_O (0.1171) in solution is significantly lower than that of H_2_O (0.3238), indicating that the D_2_O solution, despite the reduction in ionic conductivity, slows down the mass transfer of electrolyte at the electrode surface, effectively inhibiting the side reactions and the formation of H_2_. Moreover, the kinetic diffusion behaviors of the electrolytes for HER (H, D) and OER (OH, OD) were studied. The lower diffusion coefficients of the D (3.724×10^2^) and OD (3.202×10^4^) make it difficult to couple with another D or OD for the further step in the HER or OER compared to the H (5.688×10^2^) and OH (3.984×10^4^). Both sluggish kinetic behavior of the D, OD, and D_2_O in the bulk phase of the electrolyte and the surface on the anode greatly restrain the side‐reaction in the aqueous battery systems. In addition to the traditional trail‐and‐error approach for the optimization of the thermodynamic behavior of materials. Our investigation has proposed the new idea that improving the behavior of the reaction kinetics is also a long‐lasting and effective route.

**Figure 2:**
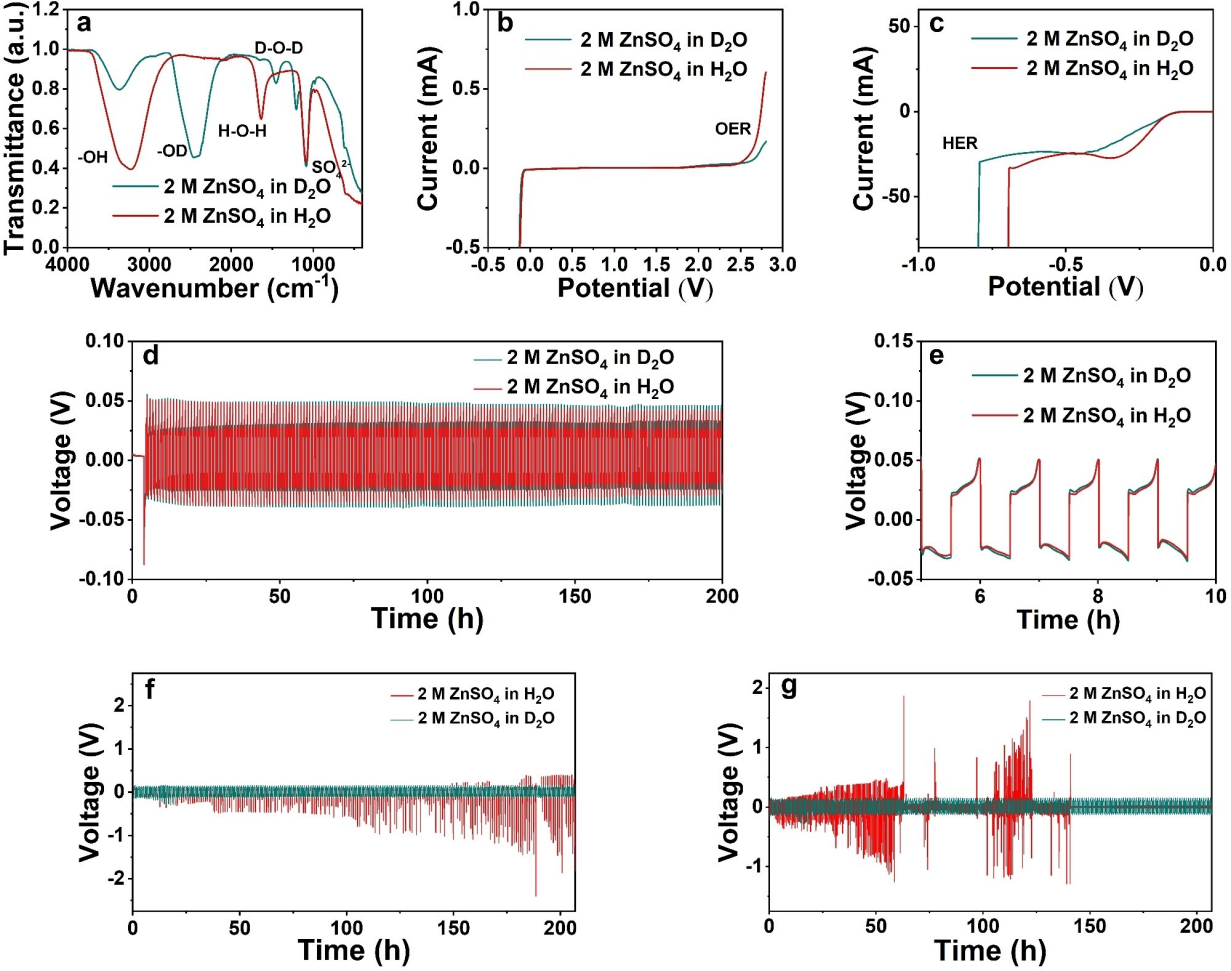
(a) FTIR spectra of 2 M ZnSO_4_ in D_2_O and H_2_O, respectively. (b) LSV test of 2 M ZnSO_4_ in D_2_O and H_2_O in Zn||Ti cells and (c) a focus on Zn deposition. Cycling performance of Zn||Zn symmetric cells at (d) 5 mA cm^−2^ for 1 mAh cm^−2^ with (e) an enlarged period of the 5^th^‐10^th^ h, (f) 20 mA cm^−2^ for 10 mAh cm^−2^ and (g) 40 mA cm^−2^ for 10 mAh cm^−2^.

The isotopic effect of hydrogen also affects the electrochemical properties of water.^[13]^ The commonly used electrolyte for ZIBs, 2 M zinc sulfate heptahydrate (ZnSO_4_ ⋅ 7H_2_O) was prepared in H_2_O and D_2_O, respectively. By observing and comparing the Fourier‐transform infrared spectroscopy (FTIR) in Figure 2a, it can be found that the D_2_O and H_2_O hydroxide ion peak positions in the electrolyte are greatly different. The FTIR spectra of ZnSO_4_ in H_2_O showed the presence of the absorption bands at 3115 and 1612 cm^−1^ for stretching and bending vibrations of H_2_O molecules, respectively.^[14]^ Correspondingly, the stretching and bending vibrations of D_2_O molecules are at 2468, 1442 and 1205 cm^−1^, respectively.^[15]^ The bands observed at 1091 cm^−1^ in H_2_O, 1201 and 1091 cm^−1^ at D_2_O indicate the asymmetric stretching mode of SO_4_^2−^_v3_.^[15]^ The absorption presented at 605 cm^−1^ indicates asymmetric bending mode of SO_4_^2−^_v4_.^[16]^ The small amount of stretching and bending vibrations of H_2_O in the D_2_O‐based electrolyte corresponds to the crystal water introduced during the preparation of the solution. As a common additive, manganese sulfate (MnSO_4_) was investigated whether it influences the absorption within the electrolyte, as shown in Figure S2a. The introduction of Mn^2+^ has little effect on the absorption behavior in the electrolyte and shows no peak shift. To investigate the electrochemical behavior of D_2_O‐based electrolyte, Zn||Ti cells were assembled to compare the EW of H_2_O and D_2_O during the Zn^2+^ deposition process. At room temperature, the ionic conductivity of D_2_O‐based electrolytes is slightly lower than that of H_2_O‐based electrolytes, which has been demonstrated in previous studies.[^3^, ^12^, ^17^] Simultaneously, the strong coordination between D_2_O and Zn^2+^ increases the proportion of coordinated water. The increase in the number of neutrons in deuterium atoms in D_2_O can significantly expand the EW of aqueous electrolytes because of the lower zero‐point energy of deuterium compounds, smaller ionic products, and larger dehydration energy of D_2_O.^[12]^ As shown in Figure 2b, 2 M ZnSO_4_ ⋅ 7H_2_O was dissolved in H_2_O and D_2_O, respectively, to explore the difference in aqueous electrolyte caused by EEI. Verified by linear sweep voltammetry (LSV) tests at a sweep rate of 10 mV s^−1^, D_2_O was able to significantly inhibit OER. As shown in Figure 2c, the potential gap of Zn deposition between D_2_O and H_2_O is only 0.02 V, indicating that it has no significant effect on the deposition potential of Zn^2+^ on metallic Zn. However, the potential of HER is significantly different between D_2_O‐ and H_2_O‐based electrolyte. The potential of HER in D_2_O is 0.1 V lower than that of H_2_O, indicating that D_2_O can inhibit HER. Based on the EEI, we found that the potential window of the ZnSO_4_ aqueous electrolyte is extended by ∼9.98 %, which can be attributed to the increase in the number of neutrons in deuterium atoms relative to protium. To further explore the effect of EEI on Zn^2+^ deposition, The Zn−Zn symmetry cell was tested by cyclic voltammetry (CV) in Figure S2b. The D_2_O‐based electrolyte exhibits a slower deposition process than the H_2_O‐based electrolyte. To analyze the cause of EEI in water solvent, on one hand, according to the principle of energy conservation, the overall energy change in the electrode reaction can be simply expressed as the Born‐Haber cycle.^[18]^ Since the activation energy of D_2_O is higher than that of H_2_O, the electrolysis of water in D_2_O is inhibited when factors such as the electron work function and hydration energy of the electrodes are considered. On the other hand, due to the non‐negligible effect of kinetics, the low diffusion coefficient of ions in the electrolyte results in a low reaction rate and reduces the reactivity at the electrolyte‐electrode interface.

As shown in Figure 2d, the D_2_O‐based and H_2_O‐based electrolytes behave similarly in the initial polarization part of the Zn||Zn symmetric cells at a current density of 5 mA cm^−2^ for 1 mAh cm^−2^, while the overpotential of the D_2_O‐based electrolyte is slightly higher than that of the H_2_O‐based electrolyte. With the increase of cycle time, from 50 h, the ZIBs with H_2_O‐based electrolyte begins to show a significant decrease in overpotential, which can be attributed to the growth of dendrites generated by the irreversible deposition of Zn^2+^ between the electrodes, resulting in a reduced overpotential.^[19]^ The D_2_O‐based electrolyte can maintain a stable overpotential value throughout the 200‐h cycle, indicating that the phenomenon of the effect of the distance between the electrodes caused by the irreversible deposition of Zn^2+^ has slowed down.^[20]^ To a certain extent, it also shows that the Zn^2+^ in H_2_O are more prone to aggregate deposition than Zn^2+^ in D_2_O. Based on the kinetic studies, due to the lower diffusion coefficient of Zn^2+^ in D_2_O, Zn^2+^ will choose a closer location to deposit rather than deposit at the tip to form a dendrite. In addition, the overpotential of the Zn||Zn symmetric cells with H_2_O‐based electrolyte exacerbates the asymmetry during cycling, which indicates that a considerable amount of irreversible deposition has occurred at the metallic Zn electrode on one side.^[21]^ As shown in Figure 2e, ZIBs with D_2_O‐based electrolyte exhibits a higher initial Zn deposition barrier, which can be attributed to the lower ionic conductivity of D_2_O and the relatively low diffusivity of Zn^2+^ in D_2_O.^[22]^ Such a difference in kinetics also leads to a slightly higher overpotential in the initial stage of the ZIBs with D_2_O‐based electrolyte than the one with H_2_O‐based electrolyte. After 50 h of cycling, the metallic Zn electrodes were taken out to compare the surface morphology, and the presence of flower‐like dendrites was still observed in Zn||Zn symmetric cell with the D_2_O‐based electrolyte, as shown in Figures S3a and b. In the Zn||Zn symmetric cell with H_2_O‐based electrolyte, a large amount of uneven ZHS covered the electrode surface, as shown in Figure S3c. However, the electrodes cycled in D_2_O‐based electrolyte showed smooth surface morphology. To examine the cycling stability of the D_2_O‐based electrolyte at high current densities, Zn||Zn symmetric cells were tested at the conditions of 20 mA cm^−2^ for 10 mAh cm^−2^ and 40 mA cm^−2^ for 10 mAh cm^−2^, as shown in Figure 2f and g. The H_2_O‐based electrolyte is difficult to cycle at such high current densities, while the D_2_O‐based electrolyte is even able to cycle for more than 200 h under the harsh condition of 40 mA cm^−2^ for 10 mAh cm^−2^. A 1 : 1 mixture of D_2_O and H_2_O was prepared as an electrolyte for further study on the Zn||Zn symmetric cell at 10 mA cm^−2^ for 1 mAh cm^−2^, as shown in Figure S4. Since D and H belong to isotopes, D_2_O and H_2_O can be infinitely miscible, which provides the possibility of flexible mixture ratio. From Figure S4, it can be clearly found that the mixing of D_2_O and H_2_O shows a more durable cycling performance and a slower overpotential reduction trend than H_2_O. Meanwhile, the electrochemical stable potential window of the electrolyte prepared by the mixture is slightly smaller than that of the pure D_2_O‐based electrolyte.

**Figure 3:**
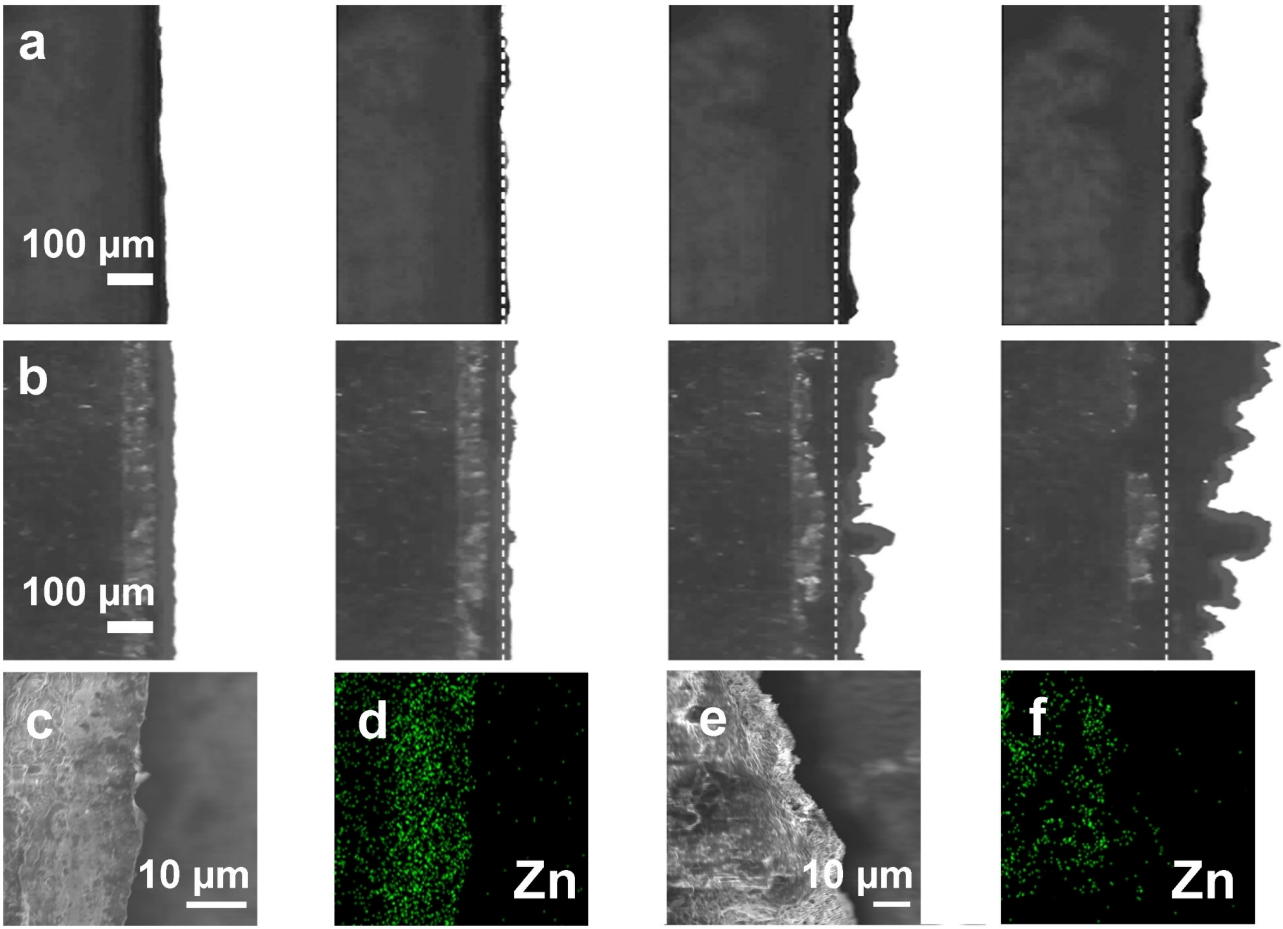
In situ optical microscope at 0, 10, 20, 30 min of electroplating process of Zn in (a) D_2_O‐based and (b) H_2_O‐based electrolytes, indicating D_2_O is able to suppress the length of Zn dendrites. (c, e) Side‐view SEM images and (d, f) corresponding EDS mapping of the metallic Zn electrode surface after 30 min deposition in D_2_O‐based and H_2_O‐based electrolytes.

Figure 3a shows the in situ optical microscopy of electroplating process of Zn at a current density of 1 mA cm^−2^ with 2 M ZnSO_4_ in D_2_O as the electrolyte. As a control, 2 M ZnSO_4_ was dissolved in H_2_O as the electrolyte for in situ optical microscopy observation under the same conditions, as shown in Figure 3b. Both for D_2_O‐ and H_2_O‐based electrolytes, Zn deposits formed on the surface layer after electroplating at a current density of 1 mA cm^−2^ for 30 min. The Zn deposition thickens significantly with increasing plating time. From the case of 10 min, thicker Zn deposition has been exhibited in the H_2_O‐based electrolyte, and Zn^2+^ are preferentially deposited on the tips of the initial protrusions. At 30 min, dendrites with a length of about 200 μm have appeared in the H_2_O‐based electrolyte. It is obvious that the Zn deposition in the D_2_O‐based electrolyte is thinner and flatter than of the one in H_2_O‐based electrolyte after 30 min. Since the Zn^2+^ is electroplated on the surface, this indicates that the D_2_O‐based electrolyte is easier to suppress the uneven Zn dendrites than the H_2_O‐based electrolyte.^[23]^ To further explore the effect of EEI on Zn electroplating, the side‐view scanning electron microscope (SEM) images of the metallic Zn electrode surface after 30 min deposition in D_2_O‐based and H_2_O‐based electrolytes are shown in Figures i and k, respectively. The metallic Zn electrode surface in the D_2_O‐based electrolyte is very smooth and uniform, indicating its excellent ability to promote uniform deposition of Zn^2+^. As shown in the energy dispersive spectrometry (EDS) mapping in Figure 3c, the Zn element can be uniformly distributed on the electrode surface. In sharp contrast, as shown in Figure 3e, the Zn electroplating in the H_2_O‐based electrolyte is not as smooth as that of D_2_O‐based electrolyte, with significant unevenness on its surface, resulting in a distinctly different light‐dark contrast in the SEM images. In addition, the distribution of Zn element, as shown in Figure 3f, also shows the inhomogeneous deposition of Zn^2+^.

**Figure 4:**
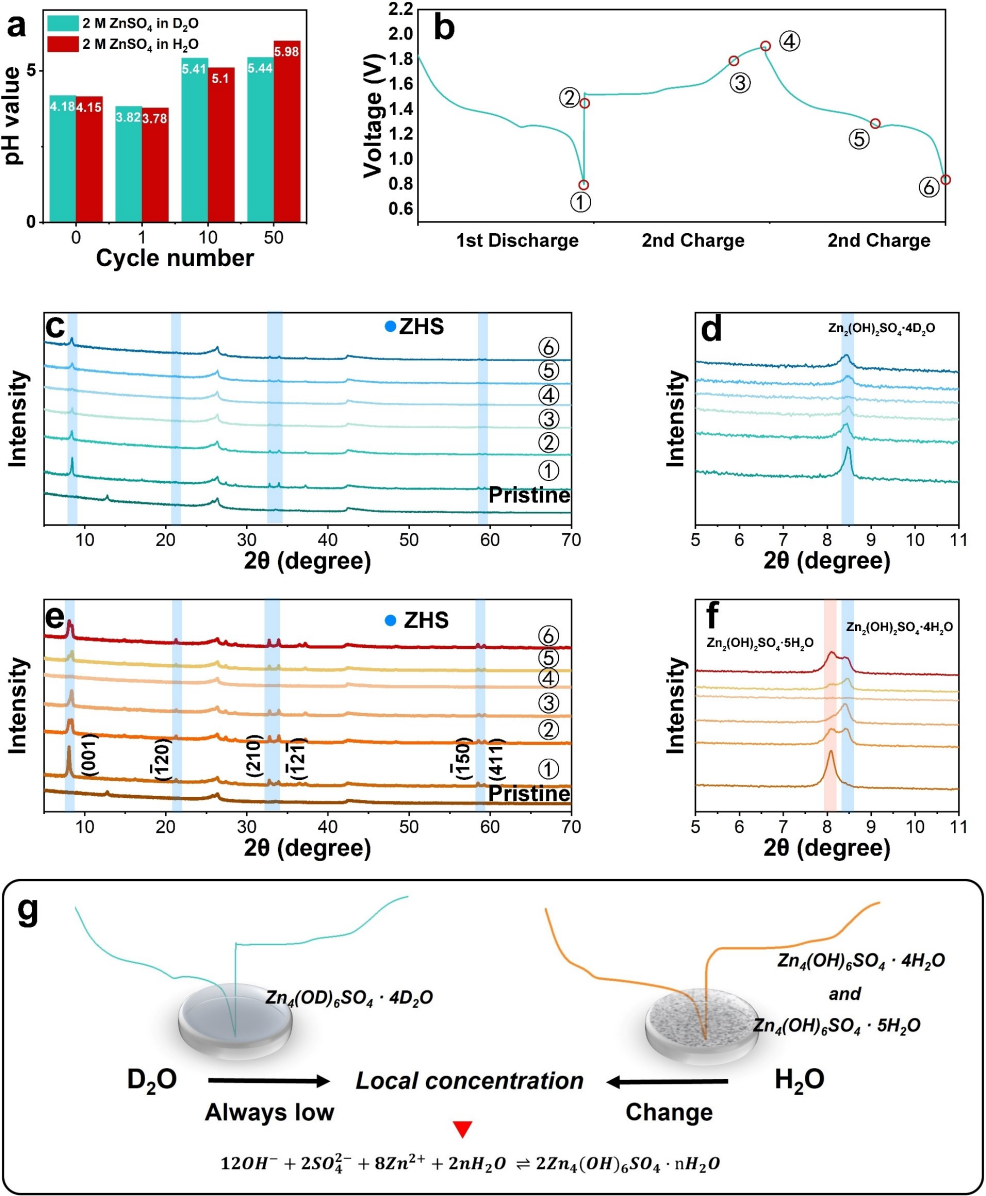
(a) In situ pH value recording of D_2_O‐ and H_2_O‐based electrolytes. (b) Different states of Ex situ XRD spectra for the MnO_2_ cathode after cycling at a current density of 2 A g^−1^ in the full cell with (c) D_2_O‐based and (e) H_2_O‐based electrolyte. The amplified plots for the highest peak (001) in (d) D_2_O‐based and (f) H_2_O‐based electrolyte. (g) Schematic diagram of the effect of D_2_O‐based and H_2_O‐based electrolytes on the formation of ZHS on the cathode surface based on the differences in local concentration.

The Zn||MnO_2_ full cells were assembled to further understand the EEI effect. Previous studies have demonstrated that the pH value of aqueous batteries can significantly affect their cycling performance, especially MnO_2_‐based ZIBs. The in situ pH values of 2 M ZnSO_4_ in D_2_O and H_2_O, respectively, were measured and incorporated in Figure 4a. The initial pH values of 2 M ZnSO_4_ dissolved in D_2_O, and H_2_O are about 4.18 and 4.15, respectively, and the difference is not obvious. However, after 10 cycles at a constant current density of 2 A g^−1^, the pH value of the D_2_O‐based electrolyte increased to 5.41, which was slightly higher than the pH value of 5.1 of the H_2_O‐based electrolytes. During the subsequent cycling, the pH value of the D_2_O‐based electrolyte was stable at 5.45, while the pH value of the H_2_O‐based electrolyte gradually increased to 6.05, which is consistent with previous studies. In ZIBs with MnO_2_ cathodes, the process of influencing pH value can be summarized in Table S2.

The EEI on the pH value change in the electrolyte is mainly due to the inertness caused by the deuterium atom.^[24]^ Since MnO_2_ is used as the cathode, the charge–discharge process of ZIBs is more understood as the intercalation and extraction of protons, Zn^2+^, and the deposition and dissolution of Mn^2+^, which is a complicated process. When an acidic electrolyte is applied, Mn^IV^O_2_ tends to react with protons (derived from water) and gain an electron to generate, Mn^III^OOH, a process corresponding to the intercalation and deintercalation of protons, as shown in Eqn. 1., ^[25]^

(1) $$2{Mn}^{III}OOH+2H^{+}\;\to {Mn}^{IV}O_{2}+\;{Mn}^{2+}+2H_{2}$$

However, as shown in Eqn. 1, Mn^III^OOH tends to continue to react with protons to form H_2_. This process irreversibly consumes protons and raises the pH value.^[26]^ When the pH value continues to rise, Zn^2+^, Mn^2+^ and OH^−^ in the electrolyte tend to form ZnMn_2_O_4_, which is less reversible, as shown in Eqn. 2.

(2) $$3{Zn}^{2+}\;6{Mn}^{2+}+24{OH}^{-}\;\vec{\leftarrow }3Zn{Mn}_{2}O_{4}+12H_{2}O\;+6e^{-}$$

The resulting ZnMn_2_O_4_ hinders the contact between the cathode and the electrolyte due to its poor electrical activity, resulting in irreversible capacity loss.^[27]^ However, because the formation of ZnMn_2_O_4_ hinders the contact between the active material and the protons in the electrolyte, the pH value of the electrolyte tends to be flat and reaches an equilibrium value. In H_2_O‐based electrolytes, this equilibrium pH is near 6. In the D_2_O‐based electrolyte, EEI makes the deuteron more inert, which alleviates the reaction of Mn^III^OOH with D^+^. On the other hand, due to the low diffusion coefficient of Zn^2+^ and OD^−^ in D_2_O, the chemical reaction slows down. However, when the pH value increases, the D_2_O‐based electrolyte attenuates the positive reaction rate in Eqn. 2 due to the lower concentration of free OD^−^ near the electrode surface, thus reaching equilibrium at a lower pH value. Therefore, from the perspective of the full cell, the D_2_O‐based electrolyte is able to well alleviate the pH value change during cycling. The reaction rate of HER and OER is reduced due to EEI, which is determined by the lower diffusion of OD^−^, D^+^ in the electrolyte and the higher reaction barrier simultaneously, reducing the production of H_2_ and O_2_.

Another major challenge of ZIBs research when MnO_2_ acts as a cathode is the generation of ZHS during cycling, and this poorly reversible precipitation hinders the effective contact between the electrode and the electrolyte.^[28]^ Many efforts have been made in the past to mitigate ZHS formation, and many electrolyte additives have been investigated.^[29]^ But this may make the researchers ignore the discussion of the solvent itself. Ex situ X‐ray diffraction (XRD) was used to compare the amount and structure of ZHS generated during cycling in D_2_O‐based and H_2_O‐based electrolytes. The different states in Figures 4c and e are marked in Figure 4b. When D_2_O was used as the electrolyte solvent in the MnO_2_‐based full cell, the formation of ZHS was significantly reduced, as shown in Figures 4c and e. To eliminate the influence of active material mass loading, temperature and other factors, it is ensured that the active material loading in the cathode is 1±0.05 mg cm^−2^ and the tested full cell is simultaneously cycled at a current density of 2 A g^−1^. The intensity ratio of the highest peak (001) of ZHS in D_2_O‐based to the carbon paper peak (∼26°) is 1.73. While the height ratio of the highest peak (001) of ZHS in H_2_O‐based to the same peak is 4.17. Due to the limitations of the test, the generation of ZHS cannot be fully quantified, but the generation of ZHS in the H_2_O‐based electrolyte is significantly higher than that in the D_2_O‐based electrolyte. More notably, as shown in Figure 4d, the ZHS generated in the D_2_O‐based electrolyte is Zn_2_(OH)_2_SO_4_ ⋅ 4H_2_O (ICDD No. 00‐044‐0673), which is caused by the lower amount of free water molecule in D_2_O.^[27b]^ For comparison, Figure 4f shows that relatively pure Zn_2_(OH)_2_SO_4_ ⋅ 5H_2_O (ICDD No. 00‐039‐0688) is generated in the H_2_O‐based electrolyte only during the first discharge. With the increase of cycle numbers, the generated ZHS mostly consists of a mixed phase composed of Zn_2_(OH)_2_SO_4_ ⋅ 4H_2_O and Zn_2_(OH)_2_SO_4_ ⋅ 5H_2_O.^[27b]^

The generation of ZHS with different structures during cycling may be an easily overlooked but an important factor affecting the cycling performance of ZIBs. As shown in Figure 4g, the phase of generated ZHS can be explained by local concentration of water molecules. When the concentration of water molecules at the electrode‐electrolyte interface is sufficient, Zn_2_(OH)_2_SO_4_ ⋅ 5H_2_O tends to be generated. In the first discharge of the full cell in the H_2_O‐based electrolyte, Zn_2_(OH)_2_SO_4_ ⋅ 5H_2_O dominates and it is difficult to detect Zn_2_(OH)_2_SO_4_ ⋅ 4H_2_O. With the progress of the cycle and the consumption of the electrolyte, the local concentration of water molecules is distributed nonuniformly at the interface between the electrode and the electrolyte, so that the mixed phase of Zn_2_(OH)_2_SO_4_ ⋅ 4H_2_O and Zn_2_(OH)_2_SO_4_ ⋅ 5H_2_O is produced at the second discharge. However, due to the strong coordination and low diffusion brought by D_2_O, the D_2_O‐based electrolyte can keep the local concentration at a low level during the charging and discharging processes. Therefore, it is difficult to find Zn_2_(OH)_2_SO_4_ ⋅ 5H_2_O on the cathode for all D_2_O‐based full cells. We believe that this suppression of the generation of different phases of the ZHS during cycling is beneficial and can provide a more stable interface between the electrolyte and electrodes.

**Figure 5:**
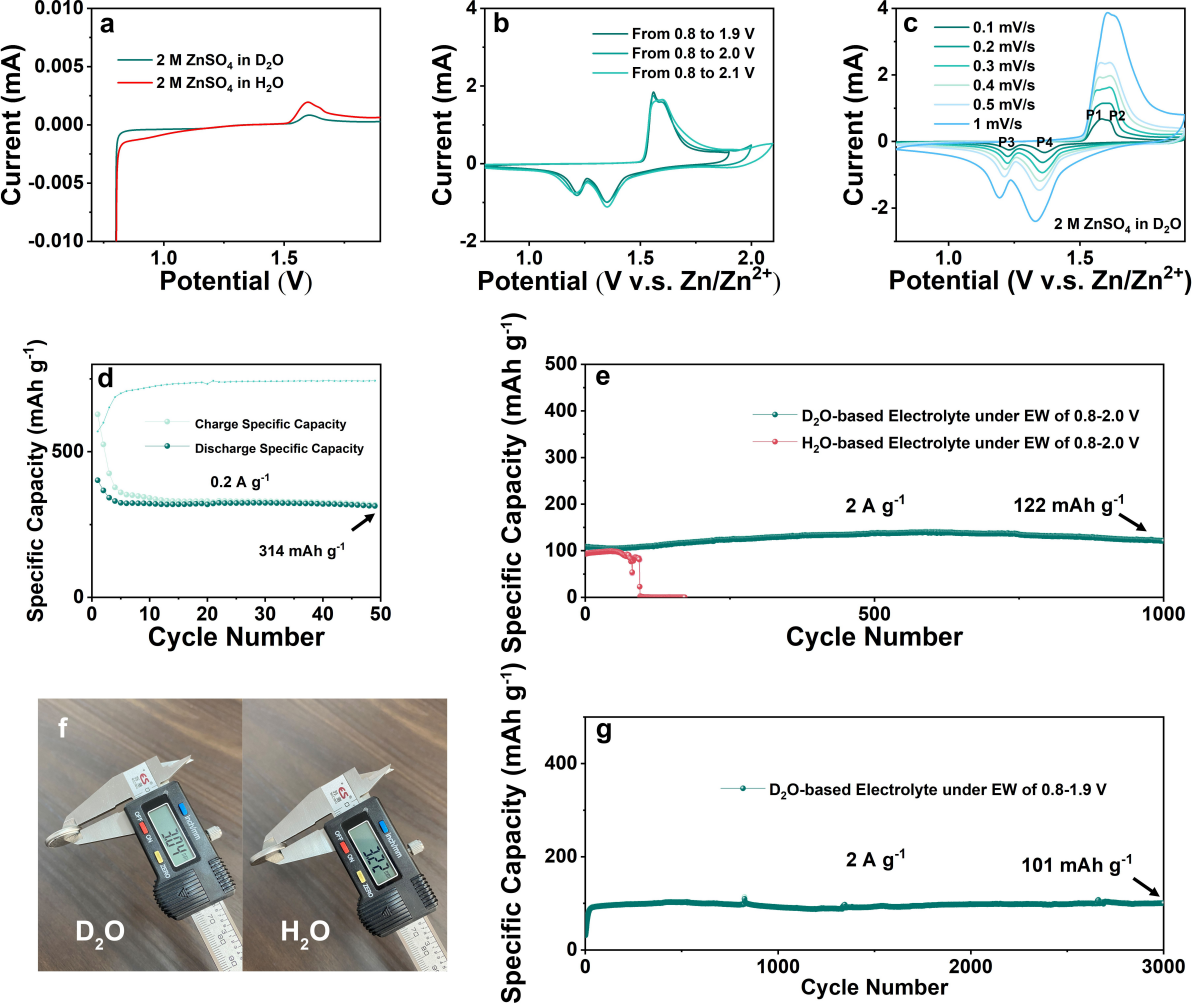
(a) LSV test of full cells of D_2_O‐ and H_2_O‐based electrolytes in the range of 0.8–1.9 V. (b) CV test of D_2_O‐based electrolyte at a scan rate of 0.3 mV s^−1^. (c) CV curves of D_2_O‐based electrolyte in the EW of 0.8–1.9 V with different scan rates. (d) Cycling performance of D_2_O‐based electrolytes in the EW of 0.8–2.0 V at a current density of 0.2 A g^−1^. (e) Long‐term cycling performance of D_2_O‐based electrolytes in the EW of 0.8–2.0 V at a current density of 2 A g^−1^. (f) The thickness comparison of full cells with D_2_O‐ and H_2_O‐based electrolytes after cycles in the EW of 0.8–2.0 V. (g) Long‐term cycling performance of the full cells with D_2_O‐based electrolyte in the EW of 0.8–1.9 V at a current density of 2 A g^−1^.

To investigate the effect of EEI on the deposition and intercalation of Zn^2+^ on the cathode in full cells, LSV tests from 0.8–1.9 V were performed in D_2_O‐based and H_2_O‐based electrolytes. As shown in Figure 5a, in the low potential region, the H_2_O‐based electrolyte performs HER more positive than the D_2_O‐based electrolyte, and the potential interval difference is ∼0.26 V. In the high potential region, the oxidation peak potentials in D_2_O‐based and H_2_O‐based electrolytes are similar, with a difference of only 0.002 V. The effect of EEI on the electrochemical performance of Zn||MnO_2_ full cells were further investigated. As shown in Figure 5b, D_2_O‐based electrolytes were applied in Zn||MnO_2_ full cells for CV tests with a scan rate of 0.3 mV s^−1^ in different potential windows of 0.8 to 1.9/2.0/2.1 V. During the charging process, two pairs of reduction and oxidation peaks were observed, the reduction peaks at 1.35 and 1.22 V, and the oxidation peaks at 1.56 and 1.61 V. The oxidation peak at 1.56 V gradually disappeared. The coin cell with D_2_O‐based electrolyte exhibited similar redox peak positions as the H_2_O‐based electrolyte (as shown in Figure S5), indicating the similarity of the two charge storage behaviors, which shows that during cycling in aqueous ZnSO_4_ electrolyte, typical MnO_2_‐based cathodic redox reactions occur.^[30]^ When the EW is extended to 0.8–2.0 and 2.1 V, the OER peak intensity of D_2_O‐based electrolyte does not show a large increase, which is quite different from the extremely strong OER generation in H_2_O‐based electrolyte under a wider potential window in Figure S5. In addition, during the cycling process of the H_2_O‐based electrolyte, the strong OER led to the change of the electrode structure, corresponding to a 0.02 V shift in the discharge peak position of the CV curve. In the D_2_O‐based electrolyte, the coincidence of the reduction peaks is still very high even after widening the EW. Cyclic voltammogram (CV) curves of the D_2_O‐based (as shown in Figure 5c) and H_2_O‐based electrolytes (as shown in Figure S6) in the normal EW of 0.8–1.9 V were measured at different scan rates of 0.1, 0.2, 0.3, 0.4, 0.5 and 1 mV s^−1^, respectively, to discuss the effect of D_2_O on the electrochemical behavior of the Zn||MnO_2_ full cells. For both D_2_O‐based and H_2_O‐based electrolyte, when the scan rate is low, two pairs of reduction and oxidation peaks were observed, with reduction peaks at 1.36 and 1.22 V and oxidation peaks at 1.56 and 1.61 V. As shown in Figure S6, the two pairs of redox peaks of the H_2_O‐based electrolyte start to overlap into a single pair from the scan rate of 0.3 mV s^−1^. As the scan rate increases, the shifts of P2 and P4 are 0.054 and 0.045 V, respectively. In Figure 5c, ZIBs with D_2_O‐based electrolyte exhibit very similar CV curves at different scan rates, which shows that their charge/discharge mechanisms are similar at high and low rates. In contrast, the shifts of P1, P2, P3, and P4 in Figure 5c are only 0.022, 0.024, 0.031, and 0.027 V, respectively, which shows a more reversible electrochemical system formed by D_2_O‐based electrolyte.

The electrochemical performance in wide EWs requires cycling validation. The electrochemical profiles of cells with an EW of 0.8–2.0 V assembled with D_2_O‐based electrolyte at a current density of 0.2 A g^−1^ are shown in Figure S7. During the charging and discharging process, the initial voltage platforms are located at about 1.55 and 1.30 V, which are consistent with the results of the CV test. As the cycle progresses, the charge and discharge platforms of the full cell assembled with D_2_O‐based electrolytes do not change significantly, indicating an extremely stable cycling performance. In the first cycle, most of the capacity in the charging process comes from the electron transferred by OER, which is caused by expanding the voltage window.^[31]^ The H_2_O‐based electrolyte cannot complete this charging process, and all H_2_O‐based full cells are constantly performing OER during the charging process. Due to the slow diffusion of ions in D_2_O, the OER is alleviated after the ion concentration on the electrode surface is reduced, allowing the full cells to continue to circulate. As shown in Figure 5d, the D_2_O‐based electrolyte exhibits stable cycling performance at EW of 0.8–2.0 V. Under the EW condition of 0.8–2.0 V, the D_2_O‐based electrolyte enables the full cells to reach a reversible capacity of 314 mAh g^−1^ after 50 cycles. The electrochemical profiles of full cells with an EW of 0.8–2.0 V assembled with D_2_O‐based electrolyte at a current density of 2 A g^−1^ are shown in Figure S6. Even if the EW is extended to 0.8–2.0 V, it still maintains a similar electrochemical behavior at the 1,000^th^ cycle, demonstrating the remarkably wide EW cycling stability of the D_2_O‐based electrolyte. As shown in Figure 5e, the full cell with H_2_O‐based electrolyte collapsed after 73 cycles at EW of 0.8–2.0 V due to the strong side reactions. The massive gas accumulation during the cycling process increases the internal pressure, and the electrodes are forced to be separated, resulting in an open circuit. In stark contrast, the full cell with D_2_O‐based electrolyte can be stably cycled for more than 1,000 cycles at EW of 0.8–2.0 V and exhibit a reversible specific capacity of 122 mAh g^−1^ at a current density of 2 A g^−1^ after 1,000 cycles. This is due to the D_2_O‐based electrolyte effectively suppressing OER and HER, as well as other aforementioned side reactions, even when the voltage is as high as 2.0 V.^[32]^ the H_2_O‐based electrolyte, with high OER and HER activity, generates a large amount of gas, causing the cathode and anode of the full cells to quickly become open circuit. The difference can be easily observed even from the appearance of the battery, as shown in Figure 5f. The D_2_O‐based electrolyte can alleviate well the gas generation problem, and the full cell still maintains close to its initial thickness after 1,000 cycles at an EW of 0.8–2.0 V. The gas generation problem of H_2_O‐based electrolyte is very serious, and it is more obvious at a wider EW. The severe outgassing caused the expansion of the coin cell casing, and its thickness changed by about 50 %, which seriously affected the stability and safety of the ZIBs. However, the low diffusion coefficient caused by EEI leads to an increase in the internal resistance of the ZIBs, as shown in Figure S7 for the EIS test of D_2_O‐based and H_2_O‐based electrolytes. In the ultra‐high frequency region (>10 kHz), in comparison to the ohmic resistance reflecting the transport of electrons and ions in the electrolyte, the D_2_O‐based electrolyte has a higher resistance (R_s_=1.41 Ω, *R*_ct_=190.34 Ω, R_L_=58.32 Ω) than the H_2_O‐based electrolyte (R_s_=1.07 Ω, *R*_ct_=140.33 Ω, R_L_=56.637 Ω), which indicates that the low‐rate diffusion behavior of ions in D_2_O. In the high frequency region, the resistance of the D_2_O‐based electrolyte is still higher than that of the H_2_O‐based electrolyte, indicating that the diffusion rate of Zn^2+^ at the interface between the electrode and the D_2_O‐based electrolyte is lower than that of the H_2_O‐based electrolyte. In the low frequency region, the straight line corresponding to the solid‐phase diffusion exhibits almost similar slopes, indicating that the EEI has no effect on the solid‐phase diffusion of ions inside the electrode. When we examine the D_2_O‐based electrolyte with the commonly used EW of 0.8–1.9 V, it exhibits an excellent cycling performance. The reversible capacity of 101 mAh g^−1^ can still be maintained after 3,000 cycles at a current density of 2 A g^−1^, and its capacity retention rate is close to 100 %. The ZIBs with H_2_O‐based electrolyte showed severe capacity decay and only displayed a specific capacity of 58 mAh g^−1^, as shown in Figure S10. When the mixture of D_2_O and H_2_O is used as the solvent for the electrolyte, D_2_O can still significantly improve the stability of the ZIBs. As shown in Figure S11, when the volume ratio of D_2_O to H_2_O is 2 : 8, the Zn||MnO_2_ full cell can exhibit a reversible capacity of 100 mAh g^−1^ after 1000 cycles at a current density of 2 A g^−1^. When the proportion of D_2_O was adjusted to 50 % and 80 %, the Zn||MnO_2_ full cells could exhibit specific capacities of 105 and 109 mAh g^−1^, as shown in Figures S12 and S13, after 1000 cycles at a current density of 2 A g^−1^.The EEI brought by D_2_O applied to aqueous ZIBs can suppress OER, HER and other side reactions during cycling, which makes the D_2_O‐based electrolyte greatly promote the cycling stability of ZIBs.

## Conclusion

In conclusion, we reveal the application of the electrochemical isotopic effect between heavy water (D_2_O) and normal water (H_2_O) electrolytes in ZIBs. Theoretical calculations show that compared with H_2_O, the ion diffusion coefficient in D_2_O is lower, which can effectively suppress the generation of side reactions kinetics. Due to the kinetic and thermodynamic differences, D_2_O‐based aqueous electrolytes exhibit a wider electrochemical window than H_2_O‐based electrolytes, showing limited electroactivity for OER/HER, less pH value change, and generation of ZHS. Through the change of bound water in ZHS during cycling, it was found for the first time that D_2_O suppresses the generation of different ZHS phases during cycling, thus providing a more stable electrode‐electrolyte interface. By using D_2_O‐based electrolyte, aqueous ZIBs with MnO_2_ cathode and metallic Zn anode exhibited a specific capacity of 122 mAh g^−1^ after 1000 cycles at a current density of 2 A g^−1^ at EW of 0.8–2.0 V, demonstrating an excellent cycle life. Due to the miscibility of D_2_O and H_2_O, and the price of D_2_O (<1.5 USD/g) is comparable to traditional electrolyte solvents/additives of ZIBs, our strategy can provide insights into the rational design and flexible production of electrolytes for aqueous batteries. Moreover, the change of ZHS in the cycling process will also be a direction worth pursuing. More importantly, our study shows that improving the reaction kinetics is an effective way to improve the stability and long‐term cycling performance of ZIBs.

## Conflict of interest

The authors declare no conflict of interest.

1

## Supporting information

As a service to our authors and readers, this journal provides supporting information supplied by the authors. Such materials are peer reviewed and may be re‐organized for online delivery, but are not copy‐edited or typeset. Technical support issues arising from supporting information (other than missing files) should be addressed to the authors.

Supporting Information

## Data Availability

The data that support the findings of this study are available from the corresponding author upon reasonable request.
