# Supplementary material for: When It's Heavier: Interfacial and Solvation Chemistry of Isotopes in Aqueous Electrolytes for Zn‐ion Batteries
Source: Angew Chem Int Ed Engl. 2023 Mar 10;62(16):e202300608. doi: 10.1002/anie.202300608 (PMC10946563; doi:10.1002/anie.202300608)
Supplement: Supplementary file 1 — Supporting Information [file ANIE-62-0-s001.pdf]

Angewandte Chemie
Eine Zeitschrift der Gesellschaft Deutscher Chemiker

Supporting Information

When It's Heavier: Interfacial and Solvation Chemistry of Isotopes in Aqueous Electrolytes for Zn-ion Batteries

*X. Gao, Y. Dai, C. Zhang, Y. Zhang, W. Zong, W. Zhang, R. Chen, J. Zhu, X. Hu, M. Wang, R. Chen, Z. Du, F. Guo, H. Dong, Y. Liu, H. He, S. Zhao, F. Zhao, J. Li, I. P. Parkin, C. J. Carmalt, G. He**

Experimental section

Materials Characterization

All chemicals are bought from Merck UK. The crystal structure was investigated via XRD (STOE SEIFERT) using Cu-Kα radiation (λ = 0.07 nm) in the range of 5° to 40° (2θ) and Mo-Kα radiation (λ = 0.15 nm) in the range of 5° to 70° (2θ). The morphologies of the samples were examined using a scanning electron microscope (SEM, JEOL-JSM-6700F) equipped with an Energy dispersive spectrometry (EDS) attachment under a low vacuum of < 3×10^{-4} Pa. The samples were coated with platinum prior to SEM analysis. Fourier-transform infrared spectroscopy (FTIR) was achieved by Attenuated Total Reflectance Fourier transform infrared spectroscopy (ATRFTIR, BRUKER, platinum-ATR). AZ9861 pH meter (AZ Instrument Corp.) was performed to monitor the pH values. The in-situ optical microscope was conducted on VisiScope® BL254 T1 (VWR) instrument with a specially designed electrolytic cell in a Zn||Zn symmetric configuration.

Electrochemical Measurement

For Zn||MnO_2 full cells, the slurry was prepared by mixing the active materials (MnO_2), carbon black (Super P, TIMCAL Graphite & Carbon), and poly(vinylidene fluoride) (PVDF, M.W. 534000, Sigma-Aldrich) at a weight ratio of 7:2:1 using N-methyl-2-pyrrolidone (NMP); the mixture was then coated on the carbon paper. After drying in a vacuum oven at 60 °C for 24 h, cathodes with ~1.5 mg cm^{-2} of the active materials were achieved. For Zn||Zn symmetric cells, metallic Zn electrodes are symmetrically loaded into the cell. CR2032 coin cells were assembled by a traditional method in an open-air system using glass-fibers (Whatman, GF/A) and metallic Zn foils as separators and anodes, respectively. Additionally, for full cell testing, 2 M $ZnSO_4$ and 0.2 M $MnSO_4$ were used as the electrolyte. For other testing, 2 M $ZnSO_4$ is prepared as the electrolyte. The coin cells were tested using a Neware battery test system (Shenzhen, China) at 25 °C. The current density and specific capacity were calculated using only the weight of the active material. CV tests were performed

using a Biologic VMP3 electrochemical workstation at 25 °C. EIS tests were performed using a Biologic VMP3 electrochemical workstation at 25 °C in the frequency range of 10^5–10^{-1} Hz.

Computation Methods

All simulations were carried out in the Materials Studio. The DFT calculations were performed in the CASTEP Module while the MD simulations were accomplished within the Forcite module.[1] In DFT calculations, the cut-off energy was set as 650 eV, the *Γ*-centered *k*-mesh was adopted as 5×5×1 for the geometry optimization for all structures. The criteria of convergence for max force, stress, and displacement were 0.02 eV/A, 0.02 GPa, and 0.001 Å, individually. The Perdew-Burke-Ernzerh of generalized gradient approximation (GGA)[2] was employed, and the ultrasoft pseudopotential for each atom was adopted.

The molecular dynamics simulations were performed with the forcite module in the Materials Studio in a canonical ensemble (NVT) at 300 K, and the temperature was maintained using the Nosé–Hoover thermostat.[3] The equations of motion were integrated with a 1 fs time step and the total simulation time was set as 1000 ps. The COMPASSII forcefield, whose parameters have been thoroughly validated using various calculation methods including extensive molecular dynamics simulations of liquids, crystals, and polymers, was adopted to describe the interactions in the system.

The adsorption energy (E_{adsor}) was calculated by the following equations[4]:

$$E_{adsor} = E_{total} - E_{sub} - E_{Li}$$

The E_{total}, E_{sub} , and E_{Li} indicates the energies of the complex system, the substrate (Zn), and H(D) or OH (OD) clusters, respectively.

The diffusion coefficients (D) of the reactants were obtained from the mean square displacements (MSD) using the relation[5]:

$$\frac{\partial[MSD(\tau)]}{\partial\tau} = 4D$$

$$MSD_{xy}(\Delta t) =< [x(t+\Delta t) - x(t)][y(t+\Delta t) - y(t)] >$$

Where the Δt indicates the time interval to obtain the mean square displacement of ions, r means the position vector. The angle brackets represent

taking an average of all times t. The x and y present that the ions migrate on the surface of the substrate.

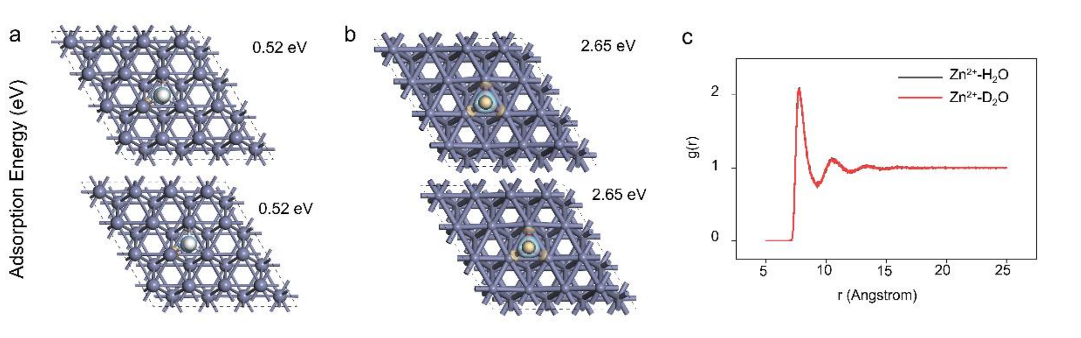

Figure S1. (a) The charge density difference when H atom and D atom are adsorbed on the metallic Zn. (b) The charge density difference when OH and OD that are adsorbed on the metallic Zn, respectively. (c) RDFs for Zn^{2+}-O (D2O) and Zn^{2+}-O (H2O) from MD simulations of electrolyte.

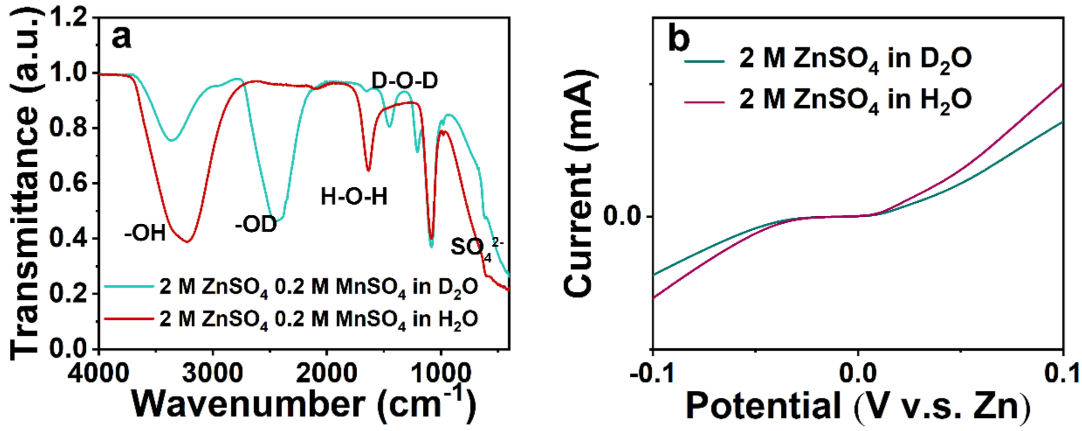

Figure S2. (a) FTIR spectrum of 2 M $ZnSO_4$ and 0.2 M $MnSO_4$ in D_2O and H_2O. (b) CV test of Zn||Zn symmetric cells of 2 M $ZnSO_4$ in D_2O and H_2O.

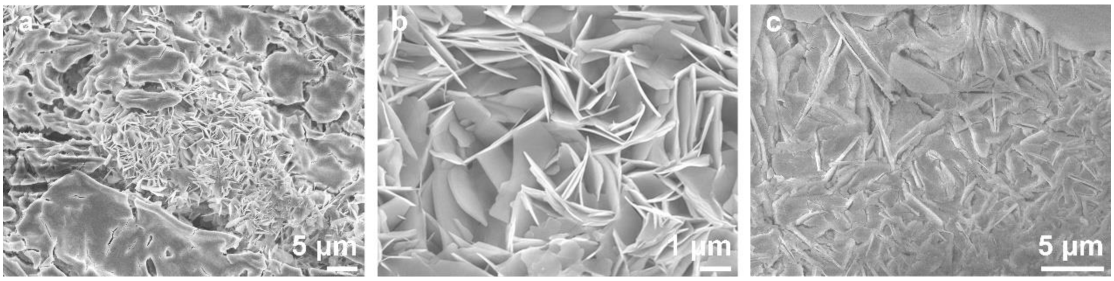

Figure S3. SEM images of (a) surface and (b) dendrite morphology on electrodes after cycling of Zn||Zn symmetric cells in D_2O-based electrolyte. (b) surface with extensive coverage on electrodes after cycling of Zn||Zn symmetric cells in H_2O-based electrolyte.

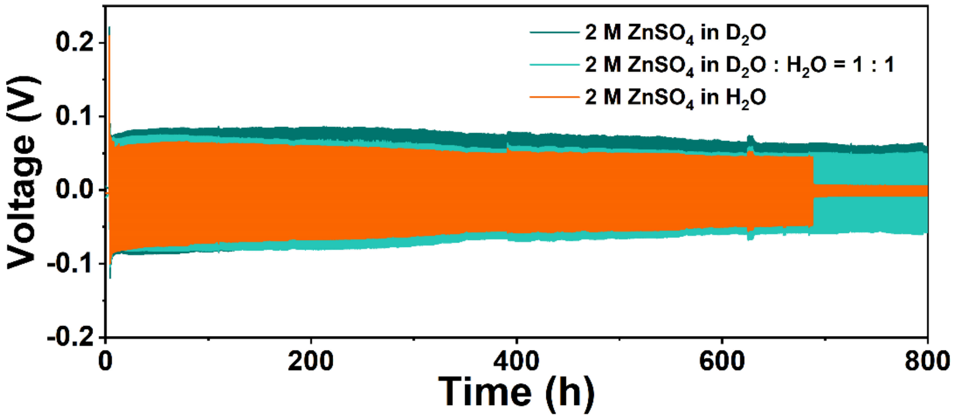

Figure S4. Cycling performance of Zn||Zn symmetric cells at 10 mA cm^{-2} for 1 mAh cm^{-2} in the electrolyte of D_2O, H_2O and a mixture of D_2O and H_2O in a ratio of 1:1.

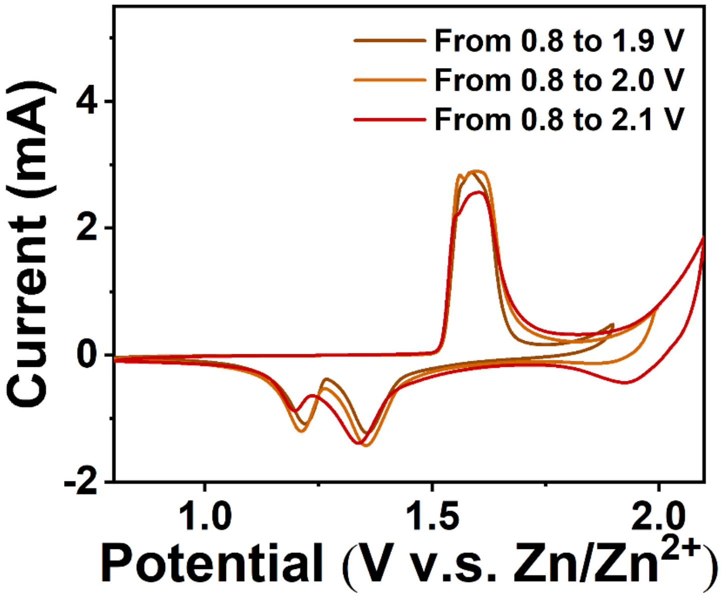

Figure S5. CV test of H_2O-based electrolyte in the EW of 0.8-1.9/2.0/2.1 V, respectively.

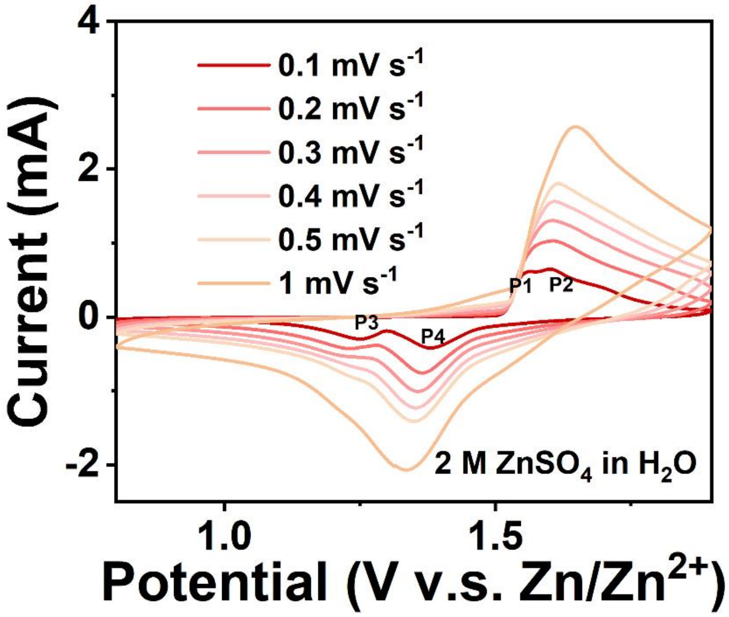

Figure S6. CV curves of H_2O-based electrolyte in the EW of 0.8-1.9 V with scan rates ranging from 0.1 mV s^{-1} to 1 mV s^{-1}.

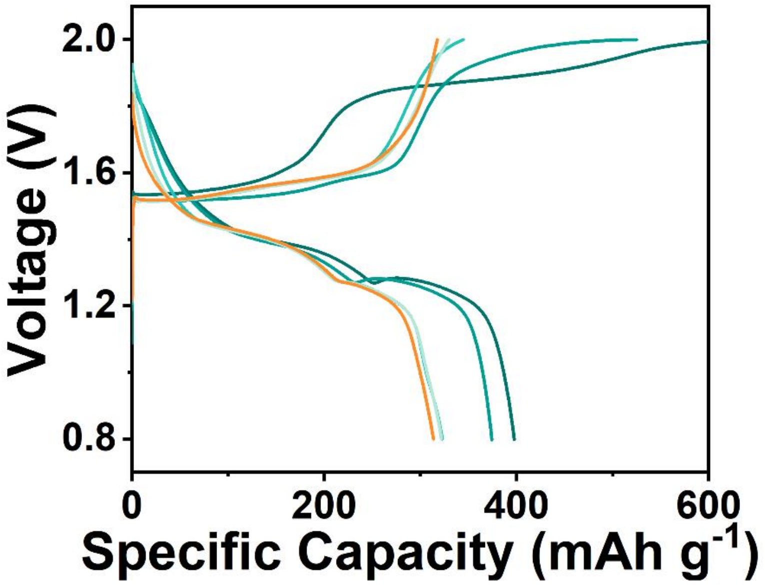

Figure S7. Electrochemical profile of full cells in the D_2O-based electrolyte in the EW of 0.8-2.0 V at a current density of 0.2 A g^{-1}.

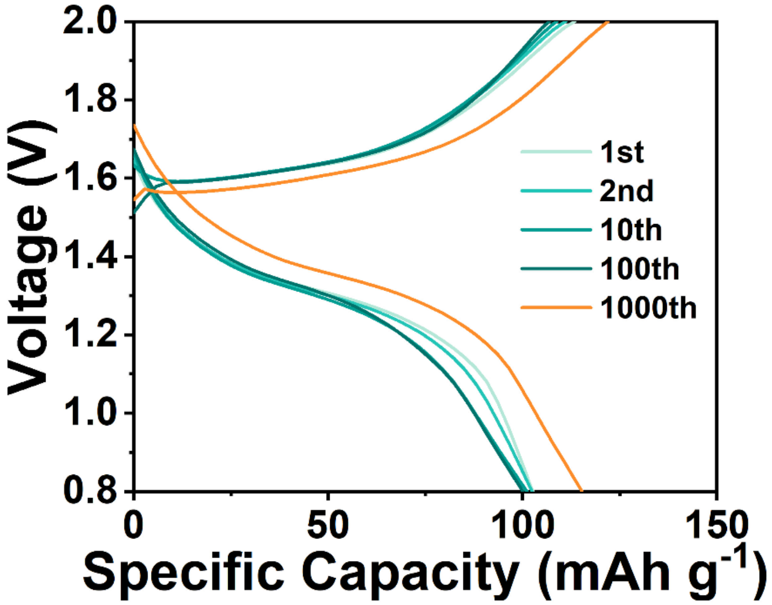

Figure S8. Electrochemical profile of full cell in D_2O-based electrolyte in the EW of 0.8-2.0 V at a current density of 2 A g^{-1}.

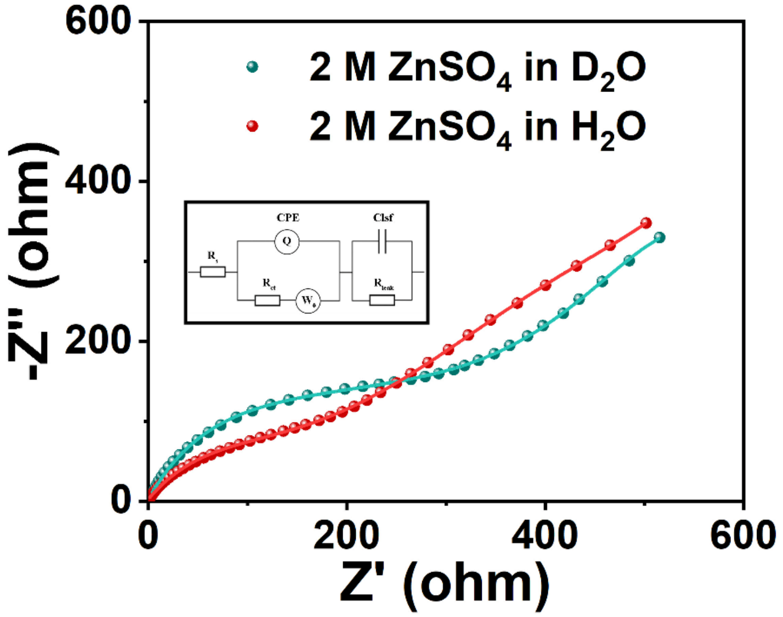

Figure S9. EIS test of full cells with D_2O- and H_2O-based electrolytes.

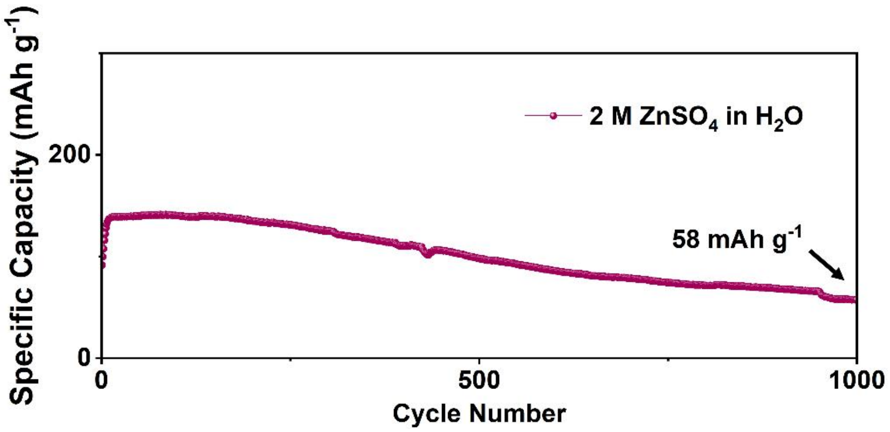

Figure S10. Long-term performance of Zn||MnO_2 full cell in H_2O-based electrolyte at a current density of 2 A g^{-1}.

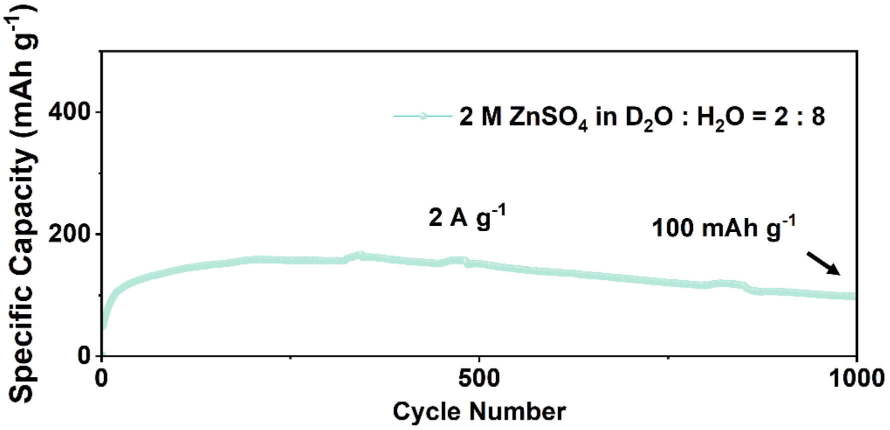

Figure S11. Long-term performance of Zn||MnO_2 full cell in the electrolyte of D_2O:H_2O = 2:8 at a current density of 2 A g^{-1}.

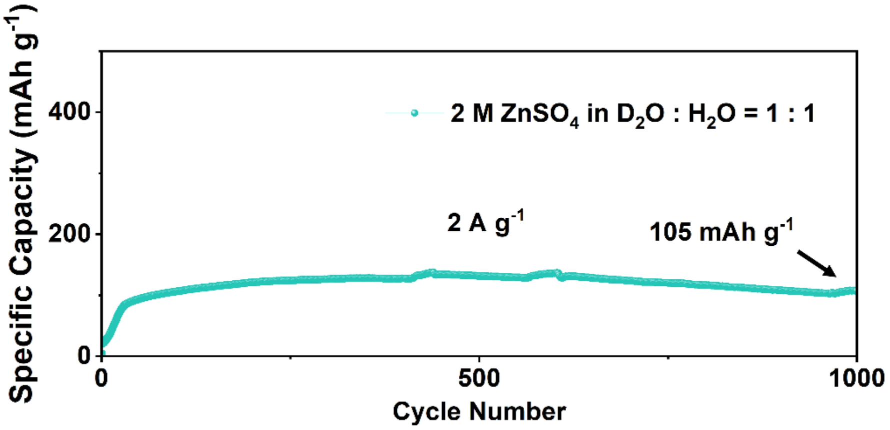

Figure S12. Long-term performance of Zn||MnO_2 full cell in the electrolyte of D_2O:H_2O = 1:1 at a current density of 2 A g^{-1}.

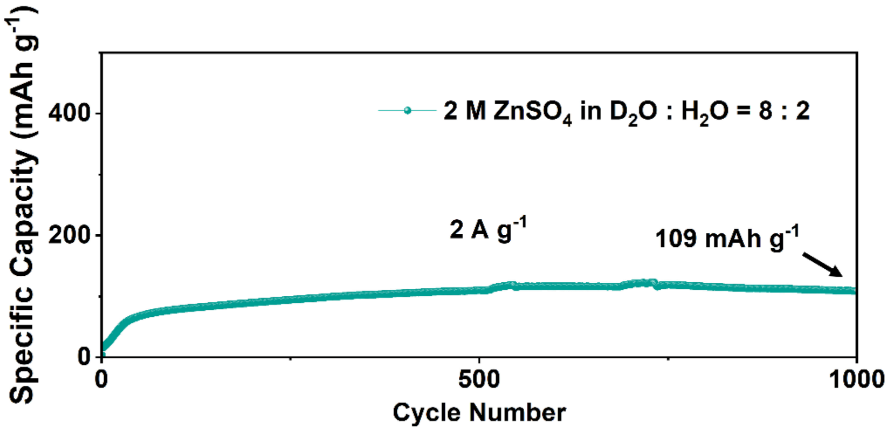

Figure S13. Long-term performance of Zn||MnO_2 full cell in the electrolyte of D_2O:H_2O = 8:2 at a current density of 2 A g^{-1}.

Table S1. Physicochemical properties of H_2O and D_2O.[6]

Physicochemical Properties	H_2O	D_2O
Density at 25 °C (g cm^{-3})	0.997	1.104
Vapor pressure at 20 °C (kPa)	2.337	2.000
Dissociation constant at 25 °C	1.00×10^{-14}	1.35×10^{-14}
Viscosity at 25 °C (cp)	0.890	1.107
Diffusion coefficient at 25 °C (cm^2 s^{-1})	2.31×10^{-5}	1.80×10^{-5}
Heat of fusion (kJ mol^{-1})	6.012	6.343
Heat of vaporization (kJ mol^{-1})	40.657	41.521
Heat of sublimation (kJ mol^{-1})	50.953	52.879
Melting point (°C)	0	3.81
Boiling point °C	100.00	101.42

Table S2. Overview of the reaction mechanisms of the ZIBs during cycling. The reactions are standardized to the transfer of 6 e$^-$, respectively.[7]

Reaction during the cycling	Discharge pH effect	Charge pH effect
Zn/Zn^{2+} dissolution (Discharge)/deposition (Charge): $3Zn \rightleftharpoons 3Zn^{2+} + 6e^- \ (E_0 = 0\,V\ vs. Zn/Zn^{2+})$		
MnO_2/Mn^{2+} dissolution/deposition in acid environment $3MnO_2 + 6e^- + 12H^+ \rightleftharpoons 3Mn^{2+} + 6H_2O$	↑	↓
ZHS precipitation: $12OH^- + 2SO_4^{2-} + 8Zn^{2+} + 2nH_2O \rightleftharpoons 2Zn_4(OH)_6SO_4 \cdot nH_2O$ $n = 4, 5$	↓	↑
HER, Charge: $6H^+ + 6e^- \rightleftharpoons 3H_2\ (E_0 = +0.52\,V\ vs. Zn/Zn^{2+} at\ pH{\sim}4)$		↑
H^+ intercalation/deintercalation: $Mn^{IV}O_2 + H^+ + e^- \rightleftharpoons MnOOH$	↑	↓
Disproportionation of MnOOH $2Mn^{III}OOH + 2H^+ \rightarrow Mn^{IV}O_2 + Mn^{2+} + 2H_2$		↑
Zn^{2+} intercalation/deintercalation: $xZn^{2+} + 2xe^- + MnO_2 \rightleftharpoons Zn_xMnO_2$		
ORR, Discharge/OER, Charge $1.5O_2 + 6H^+ + 6e^- \rightleftharpoons 3H_2O$ $(E_o = +1.75\,V\ vs. Zn/Zn^{2+} at\ pH{\sim}4)$	↑	↓
$ZnMn_2O_4$ formation after few cycles (Charge): $3Zn^{2+}\ 6Mn^{2+} + 24OH^- \rightleftharpoons 3ZnMn_2O_4 + 12H_2O + 6e^-$		↓

Reference

[1] S. J. Clark, M. D. Segall, C. J. Pickard, P. J. Hasnip, M. I. J. Probert, K. Refson, M. C. Payne, *Z. Kristallogr. Cryst. Mater.* **2005**, 220, 567.

[2] J. P. Perdew, K. Burke, M. Ernzerhof, *Phys. Rev. Lett.* **1996**, 77, 3865.

[3] S. Nosé, *Mol. Phys.* **2006**, 52, 255.

[4] C. Zhang, Y. Dai, Q. Sun, C. Ye, R. Lu, Y. Zhou, Y. Zhao, *Mater. Today Adv.* **2022**, 16, 100280.

[5] M. S. Green, *J. Chem. Phys.* **1952**, 20, 1281.

[6] a) J. Chou, Y. Zhao, X. T. Li, W. P. Wang, S. J. Tan, Y. H. Wang, J. Zhang, Y. X. Yin, F. Wang, S. Xin, *Angew. Chem., Int. Ed.* **2022**, e202203137; b) M. Jelinska-Kazimierczuk, J. Szydlowski, *J. Solution Chem.* **2001**, 30, 623; c) X. Zhou, L. Wang, X. Fan, B. Wilfong, S.-C. Liou, Y. Wang, H. Zheng, Z. Feng, C. Wang, E. E. Rodriguez, *Chem. Mater.* **2020**, 32, 769.

[7] a) B. Lee, H. R. Seo, H. R. Lee, C. S. Yoon, J. H. Kim, K. Y. Chung, B. W. Cho, S. H. Oh, *ChemSusChem* **2016**, 9, 2948; b) O. Fitz, C. Bischoff, M. Bauer, H. Gentischer, K. P. Birke, H. M. Henning, D. Biro, *ChemElectroChem* **2021**, 8, 3553; c) A. Bayaguud, X. Luo, Y. Fu, C. Zhu, *ACS Energy Lett.* **2020**, 5, 3012.
